# Heat-Stable Enterotoxins of Enterotoxigenic *Escherichia coli* and Their Impact on Host Immunity

**DOI:** 10.3390/toxins11010024

**Published:** 2019-01-08

**Authors:** Haixiu Wang, Zifu Zhong, Yu Luo, Eric Cox, Bert Devriendt

**Affiliations:** 1 Laboratory of Immunology, Department of Virology, Parasitology and Immunology, Faculty of Veterinary Medicine, Ghent University, Salisburylaan 133, 9820 Merelbeke, Belgium; Haixiu.Wang@UGent.be (H.W.); Eric.Cox@UGent.be (E.C.); 2 Laboratory of Gene Therapy, Department of Nutrition, Genetics and Ethology, Faculty of Veterinary Medicine, Ghent University, Heidestraat 19, 9820 Merelbeke, Belgium; Zifu.Zhong@UGent.be; 3 Animal Medical Testing Center, Department of Animal Production, Faculty of Agricultural & Biological Engineering, Jinhua Polytechnic, No. 888 Haitang West Street, Jinhua 321007, China; luoyu@jhc.cn

**Keywords:** ETEC, heat-stable enterotoxins, vaccination strategies

## Abstract

Enterotoxigenic *Escherichia coli* (ETEC) are an important diarrhea-causing pathogen and are regarded as a global threat for humans and farm animals. ETEC possess several virulence factors to infect its host, including colonization factors and enterotoxins. Production of heat-stable enterotoxins (STs) by most ETEC plays an essential role in triggering diarrhea and ETEC pathogenesis. In this review, we summarize the heat-stable enterotoxins of ETEC strains from different species as well as the molecular mechanisms used by these heat-stable enterotoxins to trigger diarrhea. As recently described, intestinal epithelial cells are important modulators of the intestinal immune system. Thus, we also discuss the impact of the heat-stable enterotoxins on this role of the intestinal epithelium and how these enterotoxins might affect intestinal immune cells. Finally, the latest developments in vaccination strategies to protect against infections with ST secreting ETEC strains are discussed. This review might inform and guide future research on heat-stable enterotoxins to further unravel their molecular pathogenesis, as well as to accelerate vaccine design.

## 1. Introduction

Enterotoxigenic *Escherichia coli* (ETEC) are a common cause of acute diarrheal disease in both humans and farm animals [1,2,3]. Children and travelers within ETEC endemic regions are the main populations that suffer from acute diarrheal illnesses [4,5]. Indeed, heat-stable enterotoxins (STs) producing ETEC strains are ranked eighth among enteropathogens leading to diarrhea with mortality in 2016, accounting for 3.2% total diarrhea with mortality among all age groups, and 4.2% in children under five years old [6,7,8]. On top of that, repeated moderate-to-severe ETEC infections in children can cause long term consequences, such as malnutrition, stunted growth, chronic inflammation of the gut and impaired cognate development [9,10,11,12]. Moreover, ETEC account for up to 70% of cases of traveler’s diarrhea, although improved hygiene has reduced the risk to 8% to 20% in some countries [2,5]. Among farm animals, ETEC infections are mainly reported in neonatal cattle and piglets. In the latter, ETEC infections during the post-weaning period increase the mortality rate and hamper growth, leading to severe economic losses for farming industries worldwide [13,14].

Enterotoxigenic *Escherichia coli* are spread via fecal–oral transmission among hosts and several virulence factors, such as adhesins and enterotoxins, play an important role in its pathogenesis. Upon ingestion and after reaching the gastrointestinal tract, ETEC colonize the small intestine through an interaction of fimbrial and non-fimbrial adhesins with specific receptors present in the apical membrane of the small intestinal epithelium [15]. To date, at least 25 distinct colonization factors have been identified in human ETEC strains, while in swine-specific ETEC strains only five different fimbrial adhesins have been identified [16,17]. For most of the fimbriae of the pig-specific ETEC strains the receptor has been identified [18]. However, for human ETEC strains, the epithelial interaction partners for their adhesins are only recently being unraveled [19]. Upon attachment to the epithelium, ETEC release heat-labile (LT) and/or heat-stable enterotoxins, that act upon intestinal enterocytes by disrupting the electrolyte homeostasis, resulting in fluid loss and eventually secretory diarrhea [15]. Studies in cell lines as well as animal models including humans revealed that both LT and ST contribute to ETEC infection [20,21]. Enterotoxin LT can be divided into LT-I and LT-II serogroups. Enterotoxin LTI has two variants isolated from human (LT-Ih) and porcine (LT-Ip) strains, which not only elicit diarrhea, but also improve the adherence of ETEC strains and other pathogens to the intestinal epithelium [22,23,24,25]. In contrast to the plasmid-encoded LT-I, the LT-II variants are encoded by chromosome and prophages and consist of three variants LT-IIa, LT-IIb, and LT-IIc enterotoxins, but seem to be only associated with diarrhea in calves (Table 1) [26,27]. Similar to LT, the ST enterotoxins display a certain heterogeneity and their functions stretch beyond their role in diarrhea induction. In the following sections we will focus on the current knowledge on the role of the heat-stable enterotoxins in ETEC pathogenesis, their impact on host immunity, and the development of vaccines targeting ST-induced diarrhea.

## 2. Heat-Stable Enterotoxins of ETEC from Human and Animal Origin

### 2.1. Genetics, Structure, and Secretion of Heat-Stable Enterotoxins

Heat-stable enterotoxins produced by ETEC are secreted peptides that can be divided in two types, STa and STb. While the latter is more virulent in animals and particularly in post-weaning pigs, the STa enterotoxin is more relevant in diarrhea induction in humans, newborn piglets and calves [28]. These peptides are encoded by two genes, estA (STI) and estB (STII), which are located on plasmids, and can be distinguished from each other by their solubility in methanol and their protease sensitivity. Enterotoxin STa is methanol soluble and protease resistant, while STb is methanol insoluble and protease sensitive. According to the host species, STa is further classified into two subtypes, known as STp and STh, which were originally isolated from swine and human ETEC strains, respectively [29]. While STp is widely found in porcine, bovine, and human ETEC strains, STh is only produced by human ETEC strains (Table 1) [30].

**Figure 1:**
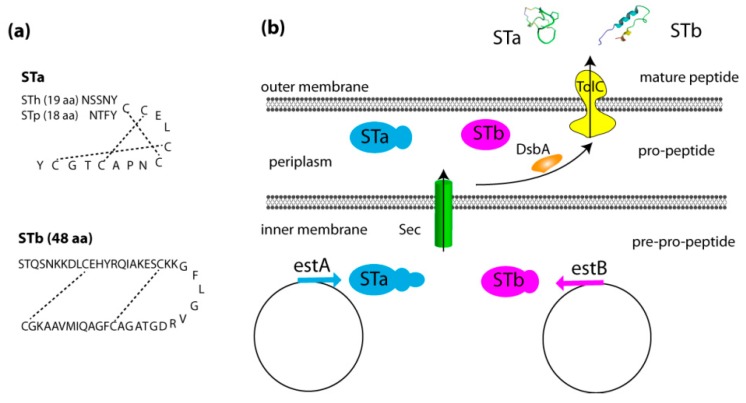
Secretion of heat-stable enterotoxins by ETEC. (**a**) The sequences of mature STa and STb peptides and the dashed lines shown in the heat-stable enterotoxin (ST) peptides represent the disulfide bonds. (**b**) Synthesis and secretion of STa and STb. Sec: Secretory pathway; DsbA: Disulfide oxidoreductase.

The STa gene encodes a 72 amino acids pre-pro-peptide precursor. Recently, six allele variants were discovered, which differ in their pro region: estA1, estA5 and estA6 from porcine origin (STp) and estA2, estA3/4 and estA7 from human origin (STh) (Table 1) [30,40]. Although the estA1 gene was first cloned from a bovine ETEC isolate, in STp^+^ strains the estA5 gene is the most common variant in isolates inducing diarrhea in animals and adults [30]. For the STh^+^ strains inducing diarrhea in children, the estA3/4 gene is the most common variant [30]. Despite these variations within the pro region, each STa allele variant is translated into a propeptide, composed of a 19 amino acids (aa) signal peptide, followed by a 34 aa prosequence, and the mature STa peptide. After translocation from the inner membrane to the periplasm, the propeptide is cleaved into the mature STa peptide (STh: 19 aa; STp: 18 aa) [28]. In the periplasm, the disulfide oxidoreductase DsbA forms three intramolecular disulfide bonds between cysteine residues Cys5-Cys10, Cys6-Cys14, and Cys9-Cys17 in STp or Cys6-Cys11, Cys7-Cys15, and Cys10-Cys18 in STh [41]. These intramolecular disulfide bridges ensure correct folding of the mature STa peptide, which closely resembles that of two mammalian peptides, guanylin and uroguanylin, and are important for its function [41]. Secretion of mature STh and STp into the extracellular environment requires the efflux protein TolC (Figure 1) [42]. Interestingly, these authors also showed that the propeptide (pro-STh) is secreted, reaffirming earlier reports on propeptide secretion by human ETEC strains [43]. This propeptide may be processed into mature STa and properly folded outside the bacteria, as the intramolecular disulfide bonds can be formed in the extracellular environment [44].

In contrast to STa, the gene encoding the heat-stable enterotoxin STb is highly conserved in ETEC isolates worldwide. Until now, only one STb allele variant has been reported (a His_12_→Asn change), which was mainly associated with STa- and Stx2-positive ETEC strains (Table 1) [38,39]. Just like STa, STb is synthetized as a 71 amino acids prepeptide, comprising a signal peptide and the mature STb enterotoxin of 48 amino acids (ca. 5.2 kDa) [45]. Once released in the periplasm, this signal peptide is cleaved to form the mature STb peptide. A correct folding of this peptide in the periplasm is mediated by DsbA, which catalyzes the formation of two disulfide bonds at position Cys10-Cys48 and Cys21-Cys36 [45]. The secretion of STb in the extracellular space is also controlled by TolC (Figure 1) [36,45]. Although sporadically reported in human ETEC strains, a role of the STb enterotoxin in human diarrheal disease is still a matter of debate [46]. In contrast, STb^+^ ETEC strains are mainly associated with diarrhea in animals and particularly in post-weaning piglets [20,47]. In the latter, using the small intestinal segment perfusion (SISP) technique, STb was shown to play a dominant role during the early secretory response as compared to the contribution of STa and LT [20]. In younger piglets (two weeks old), LT was reported to be a more important virulence factor as compared to STb [20,48,49].

### 2.2. Molecular Mechanisms of STs Induced Diarrhea

**Figure 2:**
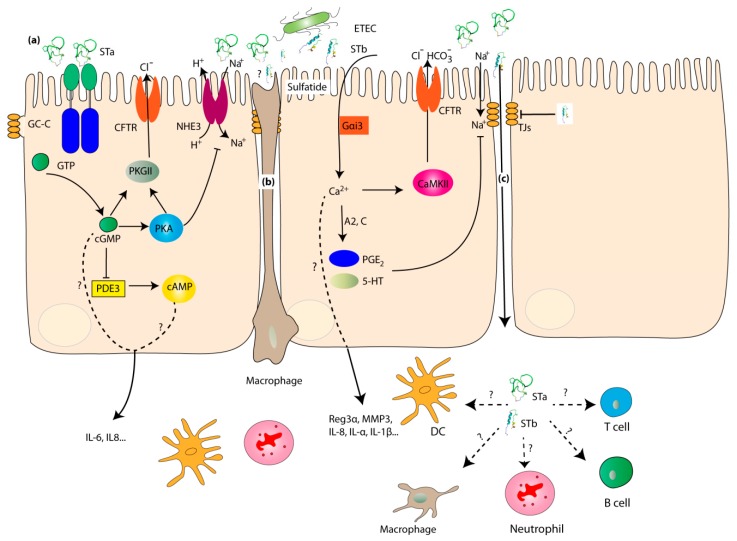
Schematic mechanisms of heat-stable enterotoxins on enterocytes and the intestinal immune system. (**a**): The impact of STs on apical membranes; (**b**): The impact of STs on transepithelial dendrites; (**c**): The paracellular transport of STs. GC-C: Guanylate cyclase C; CFTR: Cystic fibrosis transmembrane conductance regulator; NHE3: Na^+^/H^+^ exchanger; PKA: cAMP-dependent protein kinase; PKGII: cGMP-dependent protein kinase II; PDE3: cGMP-inhibitable phosphodiesterase 3; Gαi3: pertussis Toxin-sensitive GTP-binding regulatory protein; A2: Phospholipases A2; C: Phospholipases C; PGE2: Prostaglandin E2; 5-HT: 5-hydroxytryptamine; CaMKII: Calmodulin-dependent protein kinase II; MMP1: Matrix metallopeptidase 1; TJs: Tight junctions.

The release of STs into the small intestine enables their binding to target receptors in the brush border membrane of the small intestinal epithelial cells, which activates intracellular signaling cascades, resulting in a disruption of the electrolyte homeostasis and finally leading to fluid secretion [15]. Heat-stable enterotoxin STa binds to the guanylate cyclase C receptor and activates its intracellular catalytic domain, causing the hydrolysis of guanosine triphosphate (GTP) and accumulation of intracellular cyclic GMP (cGMP) levels. These increased cGMP levels activate cGMP-dependent protein kinase II (PKGII) [15,50,51]. In addition, cGMP was shown to inhibit phosphodiesterase 3 (PDE3), leading to the activation of cAMP-dependent protein kinase A (PKA) [52]. Activated PKGII and PKA phosphorylate and open the cystic fibrosis transmembrane conductance regulator (CFTR) Cl^−^ channel, inducing Cl^−^ and HCO_3_^−^ release into the intestinal lumen [50,51,52,53]. Protein kinase A also phosphorylates the sodium/hydrogen exchanger 3 (NHE3) that inhibits Na^+^ reabsorption (Figure 2) [54].

Heat-stable enterotoxin STb, on the other hand, was shown to interact specifically with sulfatide present on the surface of intestinal epithelial cells in the porcine jejunum [55,56]. This interaction activates a pertussis toxin-sensitive GTP-binding regulatory protein (Gαi3) and subsequently causes a calcium ion influx through a receptor-dependent ligand-gated Ca^2+^ channel [56]. The elevated intracellular Ca^2+^ concentration in response to STb is involved in the activation of calmodulin-dependent protein kinase II (CaMKII) through the Ca^2+^-calmodulin pathway and also in the protein kinase C (PKC)-mediated activation of CFTR, resulting in fluid accumulation in the intestine [57,58,59]. Intriguingly, using ligated small intestinal loops, an inverse relationship between STb secretion and F4^+^ ETEC adhesion was reported, inciting the authors to speculate that STb-induced diarrhea is required for ETEC transmission [60]. The increased intracellular Ca^2+^ concentration was also linked to the production of the intestinal secretagogues prostaglandin E2 (PGE_2_) and 5-hydroxytryptamine (5-HT) by regulating the activity of the phospholipases A2 and C (Figure 2) [61,62].

### 2.3. Impact on Enterocytes and the Intestinal Immune System

In addition to triggering diarrhea through the mechanisms described above, heat-stable enterotoxins have multiple functions that stretch beyond this known role. For instance, the STb enterotoxin is able to increase the permeability of the intestinal epithelium by modulating the tight junctional complexes (Figure 2) [63]. Two mechanisms have been described by which STb affects tight junctions. On the one hand, STb was shown to decrease the expression of the tight junction (TJ) proteins zona occludens-1 (ZO-1) and occludin [64,65]. On the other hand, the elevated intracellular Ca^2+^ levels in response to STb redistribute claudin-1, a transmembrane protein pivotal to maintain TJ integrity, from the plasma membrane to the cytosol, leading to an increased paracellular permeability [64,65].

In addition, ST enterotoxins might also modulate innate immune responses. Using enterotoxin-deficient ETEC mutants and the porcine SISP technique to elucidate changes in the transcriptional landscape, it was shown that ETEC infection triggers a general anti-bacterial response in the small intestinal tissues through the upregulation of Reg3α, matrix metallopeptidase 1 (MMP1) and the chemokine IL-8 [20]. In addition, a STb-specific response was identified, comprising matrix metallopeptidase 3 (MMP3) and immune-related genes, like IL-17A, IL-1α, and IL-1β [20]. STa on the other hand enhanced the luminal secretion of pro-inflammatory cytokines and chemokines, like IL-6 and IL-8, in the small intestine (Figure 2) [48]. The cellular source of these upregulated genes and proteins remains unknown, but both intestinal epithelial cells and innate immune cells might account for the observed changes.

In contrast to known effects of STs on intestinal epithelial cells, to the best of our knowledge nothing is known on the impact of these enterotoxins on the function of innate (neutrophils, macrophages) and adaptive immune cells (T and B cells) residing within the villus epithelium. Given the long-lasting effects of ETEC-induced diarrhea on gut health, it might be worthwhile to investigate this.

### 2.4. STs-Based Vaccines to Combat Human and Animal ETEC Induced Diarrhea

It is beyond doubt that vaccine design has benefited from the omics revolution and the development of bioinformatics to analyze the resulting data sets. However, discussing these technologies is out of the scope of the review. In our opinion, vaccine design to prevent ETEC infection has focused on three strategies. The first strategy included ETEC colonization factors and the enterotoxin LT. The most recent vaccine candidate based on this strategy is the Etvax vaccine, which is now being tested in phase I/II clinical trials [66,67]. A second vaccine strategy is based on inducing ST neutralizing antibodies [68,69]. A third strategy was recently developed and focuses on the inclusion of conserved ETEC proteins as vaccine antigens [70].

As mentioned above, ETEC cause considerable mortality and morbidity in young children and piglets [8,14]. In contrast to piglets, which can be protected by a live oral vaccine (Coliprotect^®^, Prevtec Microbia Inc., Saint-Hyacinthe, QC, Canada), comprising a mixture of F4+ and F18+ *E. coli*, currently no vaccines are licensed to protect against human ETEC infections [71]. Currently, vaccine design to prevent ETEC infections in humans focuses on three strategies, which aim to induce protective antibodies against colonization factors, the heat-stable enterotoxins or more recently conserved ETEC antigens [69,70]. Based on our understanding of the molecular pathogenesis of ETEC, initial vaccine development focused on including colonization factors and the enterotoxin LT. However, the development of these vaccines has been hampered due to the large heterogeneity in colonization factors (CFs) of human ETEC strains [72]. In addition, a considerable amount of the human ETEC strains simultaneously express more than one CF [72]. Since ETEC strains producing any of these CFs combined with either LT and/or STa enterotoxins can cause diarrhea, an effective vaccine should induce protective immunity against all CFs and both enterotoxins. Therefore one vaccine candidate, currently in phase I/II clinical trials, contains four inactivated recombinant *E. coli* strains, which overexpress CFA/I, CS3, CS5 and, CS6, and a recombinant cholera toxin B-subunit (CTB), in which seven amino acids have been replaced by the corresponding amino acids of LT B-subunit (LTB) (ETVAX^®^, Scandinavian Biopharma, Turku, Finland) [67]. This vaccine candidate however does not contain a STa toxoid. This vaccine candidate however does not contain a STa toxoid. Additional vaccine design has now shifted to other ETEC antigens and the inclusion of ST or their toxoids, especially since ST-producing ETEC are commonly associated with severe diarrheal illness in young children in endemic areas [5,68].

However, both STa and STb are small peptides which are poorly immunogenic and display toxicity that hinders their inclusion as antigens in vaccines. To increase the immunogenicity of STs, a recombinant fusion protein comprising STp, LTB and STb (STp-LTB-STb, SLS) was generated and included in a multivalent vaccine together with F4ac and F5 antigens derived from porcine ETEC strains [68]. Upon intramuscular injection of this vaccine to sows, their offspring was passively protected against ETEC infection [73]. To reduce toxicity, mutations should be introduced without affecting the presence of epitopes, necessary for neutralizing antibodies, and at the same time avoiding cross reactivity to guanylin and uroguanylin [74]. A double mutated STh (L9S/A14T) was developed without measurable toxicity as compared to native STh. This mutant might be a good candidate to include in future vaccines [75]. To increase the immunogenicity of this, and other ST mutants showing less toxicity, they could be chemically conjugated or genetically fused to carrier proteins, such as bovine serum albumin (BSA) or LTB [76,77]. In order to broaden enterotoxin immunogenicity and design efficient ETEC vaccines, Zhang et al. developed the concept of multi-epitope fusion antigen (MEFA) to express fusion proteins that carry different antigenic elements of ETEC toxins [78,79]. This concept was first investigated using a fusion protein containing mutated porcine LT toxoid (pLT_192_) and STa (pSTa_12_ or pSTa_13_) toxoids, which retained their immunogenicity but reduced their toxicity [78]. Their results showed that immunizing sows triggered detectable anti-LT and anti-STp serum antibodies and partially protected piglets through passive immunity against an STp^+^ ETEC challenge infection [78]. Recently, they engineered a new MEFA construct composed of a mutated STp toxoid, STb, Stx2e epitopes and the A1 peptide of mutated LT toxoid (LTR_192G_-STb-Stx2e-STa_P12F_, LTR_192G_-STb-Stx2e-3xSTa_P12F_) [80]. After intraperitoneal injection of the latter fusion protein carrying three copies of STp, it induced a significantly higher anti-STp antibody titer in mice [80]. Interestingly, intramuscular immunization of gilts with this vaccine candidate adjuvanted with dmLT resulted in the passive protection of piglets against STp^+^, STb^+^, and LT^+^ recombinant ETEC challenge infections [80]. This MEFA construct was further modified to include colonization factor antigens (CFA) from human ETEC strains (MEFA CFA/I/II/IV-3xSTa_N12S_-mnLT_R192G/L211A_) [81]. This vaccine candidate triggered significant anti-adhesin and anti-toxin antibody levels in mice and pigs and protected 76.5% of the piglets against STa^+^ or LT^+^ ETEC induced diarrhea [81].

Mucosal vaccination is the most efficient way to protect against enteric infectious diseases as it induces local immune responses at the site of infection [82]. As mentioned above, ETEC pathogen diversity has slowed vaccine design. To overcome this diversity, recent research efforts using comparative genomics have identified novel conserved antigens, which are recognized by the human immune system in controlled human infection models [70,83,84] These novel antigens should be evaluated as potential vaccine candidates to prevent ETEC infections upon mucosal administration. However, the development of effective oral vaccines still encounters multiple challenges, such as antigen degradation in the gastrointestinal tract and low uptake of intact antigens by the epithelial barrier [85]. In recent years, advances in nanotechnology allowed the design of nanoparticle-based vaccines, which might overcome the bottleneck of low antigen delivery and serve as alternative oral antigen delivery systems [86,87]. Antigen-loaded nanoparticles can be taken up by specialized intestinal epithelial cells, M cells, present in the epithelium covering the Peyer’s patches, and transcytosed through this epithelium to be phagocytosed by antigen-presenting cells (APCs) residing within the basolateral M cell pocket [88]. Additionally, muco-inert polymers and bile-acid conjugation might help the nanoparticles to penetrate the mucus barrier [89,90]. These features could allow nanoparticle-based vaccines to elicit mucosal immune responses [91]. Poly (lactic-co-glycolic acid) (PLGA) and cationized gelatin nanoparticles have been selected for encapsulation of STa in a mouse model [92,93]. Future investigations need to validate this in large animal models, like piglets, as mice are not a natural host for human/porcine ETEC strains. Although nanoparticle-based ETEC vaccine candidates show many promising advantages to carry multiple ETEC derived antigens, the selection of suitable encapsulation carriers and formulating efficient nanoparticles able to elicit strong mucosal immune response remains a challenge in further investigations.

## 3. Conclusions

Enterotoxigenic *Escherichia coli* (ETEC) infections are an important cause of diarrhea in travelers, children under the age of five years, neonatal farm animals and post-weaning piglets. Most ETEC strains produce STs that are pivotal to the induction of secretory diarrhea as well as modulate the expression of pro-inflammatory cytokines, chemokines and other immune-related genes. The mechanism of action of these enterotoxins in diarrhea induction has been thoroughly elucidated, however, their impact on the immune function of enterocytes and intestinal immune cells is lacking. Further research is warranted to elucidate if, and how, heat-stable enterotoxins affect the function of these cells. This will deepen our understanding of ETEC pathogenesis and might assist in vaccine development. Indeed, given the mortality and long-term consequences of ST+ ETEC infections, there is an urgent need to develop efficacious vaccines protecting against ETEC. Strategies involving mutating STs to reduce their toxicity and genetic fusions to enhance their immunogenicity have been widely used to develop efficient ST-based ETEC vaccines in animal models. The advances in nanotechnology might allow the design of alternative ST-based vaccines to increase vaccine efficacy. These results encourage further research on ST as a vaccine target. Although these vaccines are still in the pre-clinical phase, they hold promise to potentially eliminate ETEC-induced diarrhea.

## Figures and Tables

**Table 1:** Enterotoxins produced by Enterotoxigenic *Escherichia coli* (ETEC).

Enterotoxins	Variants	Encoding Gene	Location of Genes	Host Specificity	Receptor	Reference
Heat-labile enterotoxin (LT)	LTIh	eltAB	plasmid	humans	GM1a	[25,31,32]
	LTIp	eltAB	plasmid	piglets	GM1a	[31,33]
	LTIIa	eltAB	chromosome, prophages	water-buffalo, humans	GD1b	[27,30,31]
	LTIIb	eltAB	chromosome, prophages	unknown	GD1a	[27,30,31]
	LTIIc	eltAB	chromosome, prophages	humans, calves	GM1a	[26,27,31]
Heat-stable enterotoxin (STa)	STp	estA1, estA5, estA6	plasmids	piglets, calves, humans	GC-C	[30,34]
	STh	estA2, estA3/4, estA7	plasmids	humans	GC-C	[30,34,35]
Heat-stable enterotoxin (STb)	STb	estB	plasmids	post-weaning pigs	sulfatide	[36,37]
	STb_H12N_	estB_C34A_	plasmids	post-weaning pigs	sulfatide	[38,39]

GM = Monosialotetrahexosylganglioside; GD = Disialoganglioside; GC-C = guanylate cyclase.
